# The Psychological Impact of Global Education Approach to SDGs. A Study on Emotions and Sustainability Attitudes of European Teachers

**DOI:** 10.3389/fpsyg.2022.926284

**Published:** 2022-07-13

**Authors:** Marco Boffi, Nicola Rainisio, Paolo Inghilleri

**Affiliations:** Social Psychology Research Group, Department of Cultural Heritage and Environment, University of Milan, Milan, Italy

**Keywords:** Global Education, SDGs, emotions, sustainability, sustainability attitudes, migration attitudes, sustainability assessment, social psychology

## Abstract

In line with the international policies, Global Education (GE) programs have been widely spread in European schools over the last 20 years, in order to promote environmental and social sustainability and the achievement of the Sustainable Development Goals. Despite this popularity, their effects on attitudes and behaviors have been poorly investigated so far, particularly for teachers. Our study addressed this research gap analyzing the psychological impact of an extensive GE project involving 1,303 teachers from 10 European countries. Relevant changes in teachers' emotional states and attitudes toward sustainability and migrations were analyzed through a pre-post experimental design. Results showed that the GE activities had wide positive effects on teachers, reducing their negative emotions after teaching, increasing their attitudes about sustainability, and mitigating negative attitudes toward migrants. No significant impacts on positive emotions have been detected. Educational and methodological implications of the applied psychological assessment are finally discussed.

## Introduction

The worldwide achievement of pro-environmental and sustainability goals was increasingly connected with the educational field over the past half century, as the link between education (in formal and non-formal settings) and the progressive spread of sustainable attitudes and behaviors in society has been gradually integrated into local and international policies, until it became a crucial point. This evolution was consistent with the progressive, although not linear, adoption of more systemic global policy models on environmental conservation, which integrated the economic, social and cultural components through the wider concept of sustainability (Caradonna, 2014). A close correlation between the educational dimension, environmental protection and behavior was first promoted on a global policy scale by the Tbilisi Declaration (UNESCO-UNEP., 1978), which included the creation of “new patterns of behavior of individuals, groups and society as a whole” among the main objectives of environmental education (EE), along with strengthening awareness, attitudes and values directed toward the biosphere preservation. Later, Agenda 21 [United Nations (UN), 1992] defined education as critical in improving the capacity of the people to address environmental issues, and indispensable in modifying people's attitudes in order to strengthen their abilities to cope with their sustainable development concerns. Accordingly, the most recent global policy frameworks [e.g., The 2030 Agenda for Sustainable Development, target 4.7; United Nations (UN), 2015] stated that education has an even more crucial role, as they no longer attributed to it the purpose of supporting individual attitudes and behaviors only, but of starting up real sustainable lifestyles at a societal level. Furthermore, new education approaches, the commitment to SDG 4 (i.e., quality education, which means ensuring inclusive and quality education for all and promoting lifelong learning) and the reconceptualization of education as a means for people's wellbeing and global development were included among the key issues for an effective sustainable agenda (UNESCO, 2016).

In parallel with the progressive integration of the educational issues into increasingly systemic policy models, also significant changes in the educational frameworks on environment have been observed. The initial approach based on Environmental Education (EE), focused on protecting and enhancing natural environments, has been integrated since the 90's by the introduction of the Education for Sustainable Development (ESD; UNESCO., 2005) framework, oriented toward a greater consideration of the economic, social and development dimensions (McKeown and Hopkins, 2010). According to Pauw et al. (2015), ESD-oriented programs have significantly contributed to increase awareness and skills toward sustainable development in students and the general population, where applied. In partial disagreement, other scholars offered less optimistic conclusions, both as regards the general diffusion of a systemic concept of sustainability (Sonetti et al., 2021; Norton et al., 2022), and for the applied monitoring methodologies (O'Flaherty and Liddy, 2018). Regarding attitudes and behaviors, the success of EE and ESD programs was also found to be closely connected to the emotional dimension. In his classic review, Iozzi (1989) stressed the need for greater integration of the emotional domain in school curricula, to encourage a greater effectiveness of environmental education. Later, this link was experimentally tested by several studies (Tsevreni, 2011; Russell and Oakley, 2016), confirming its efficacy. More in general, Pooley and O'Connor (2000) pointed out the crucial role of emotions in developing pro-environmental attitudes, independently and in synergy with beliefs. Accordingly, some scholars (Thomas et al., 2009; Robina-Ramírez et al., 2020) have emphasized the effectiveness of environmental and prosocial emotions in promoting concrete actions of sustainable transformation through *ad hoc* educational programs.

Although the ESD approach has also been criticized for its anthropocentrism (Kopnina, 2014), research and educational activities in this field have been found (Ardoin et al., 2013) to gradually evolve toward a growing consideration for the community dimensions and for the intersections between ecological and social issues. In this scenario, the Global Education (GE) approach to sustainability has recently emerged, in response to the risk of an excessive focus on purely ecological issues, unrelated to a proper advancement of social justice and democracy (Scheunpflug and Asbrand, 2006). GE faced a long evolution since its origins (see Hanvey, 1976), giving origin to a rich debate on its definition and the resulting educational approaches. Pike and Selby (1995) proposed a four-dimensional model which stresses GE's holism, as the educational process takes place in the interaction between four dimensions: spatial, psychological (inner), temporal and issue-related. Similarly, Long (2013) advanced a model consisting of three main dimensions: epistemic (systemic perspective and interconnectedness), psychological (connecting world and personal identity) and civic (global citizenship and local action). The Maastricht Declaration, drafted by the Council of Europe (2002), stated that “Global education is education that opens people's eyes and minds to the realities of the globalized world (...). Global education is understood to encompass Development Education, Human Rights Education, Education for Sustainability, Education for Peace and Conflict Prevention and Intercultural Education; being the global dimension of Education for Citizenship.” Later, a review (Young, 2010) of the existing GE projects argued that various approaches coexist under this label, mainly focused on interculture, civic action and ecology respectively. Accordingly, Ferguson Patrick et al. (2014) stated that GE can be conceived as an approach aiming to enable youth to participate in creating a shared future for the world, by highlighting the strong interdependence of the human community. Hence GE implies the valorization of cultural diversity, human rights and sustainable development: the environmental issues are considered as integrated in a broader societal framework. A good summary of the GE principles was provided by the guidelines of the Council of Europe (2008), updated in 2019), as eight methodological pillars (e.g., holism, problem-orientation, citizens' participation) and seven main issues, including Environmental Education, have been highlighted. Some scholars consider such holistic view of GE consistent with a shift from an anthropocentric philosophy to a biocentric philosophy, which establishes a life-centered perspective emphasizing the intertwining between humans and the environment as “the culture is in the final analysis grounded in nature” (Selby, 2000, p. 89). According to this approach, including the cultural dimension in GE means recognizing the link existing among all forms of oppression, being it toward human or not-human forms of life (e.g., racism, classism, speciesism), which reinforce each other (Gaard, 1993). The relevance of the cultural factors for GE goes back in time, as international understanding (i.e., accurate knowledge of other cultures to favor friendly relationships among countries) and multicultural education (i.e., sympathetic comprehension of the different cultures in current societies, increased by migrations) can be considered among the predecessors of GE (Fujikane, 2003). Such issues, promoted since the 40's and the 60's respectively, gradually lost popularity at both national and international levels and reached an impasse which prevented them from becoming key issues in shaping school curricula. However, since the 90's the emphasis on the more complex notion of global dynamics, rather than international, and the need to stress the interdependency also in the ecological field favored the integration of cultural issues into the wider GE approach. The relevance of cultural factors for sustainability education led to the notion of intercultural sustainability: intercultural communication and relations are seen “as an essential dimension of the discourse on global sustainability” (Busch, 2016, p. 63), including various fields such as migrations, language teaching or conflict management.

Despite the widespread popularity of educational projects inspired by the GE framework, few research have so far systematically investigated its impacts on the competences of students, and even less on teachers. Regarding students, a substantial convergence of scholars on positive impacts can be highlighted, despite the variety of contexts and applied methodologies. Research (DeNobile et al., 2014) on a sample of Australian students involved in GE-based courses found significant pre-post changes in values and attitudes on some specific dimensions: personal identity (strongest impact), social justice, community membership, environmental sustainability. Conversely, no significant changes were detected on other factors (e.g., empathy, shared emotional connection). Focusing on attitudes toward international development and sustainability, a UK survey (Hogg and Shah, 2010) was conducted by comparing the responses of those who had GE-based learning at school and those who did not. Results showed that the former are considerably more inclined to give a positive contribution to change through social actions, more comfortable with racial and religious diversity, more concerned with sustainability and poverty issues.

Less attention was devoted to the effects of GE on teachers. Most of the studies focused on the motivational factors that lead them to choose such an approach (Biberman-Shalev, 2021) and on the importance that they attribute to it within the students' school curricula (Holden and Hicks, 2007). Another well-covered topic is related to the teachers' training needs, as significant deficiencies in this regard were often highlighted (Mundy and Manion, 2008; Reysen and Katzarska-Miller, 2018). In this regard, it is also relevant to mention the growing body of research on “teaching sensitive issues,” as it often underlines the perceived lack of preparation by teachers in managing the social and ideological conflicts inherently linked with controversial topics, as those usually addressed by GE (Kello, 2016; Savenije et al., 2019). Previous studies therefore focus on the systemic or antecedent aspects of GE-based educational activities, while, to our best knowledge, no research has so far been focused on the impact of the latter on teachers' attitudes toward sustainability and other concepts related to GE. Furthermore, despite many studies on the psychological state of teachers during teaching in general (e.g., Beer and Beer, 1992; Postareff and Lindblom-Ylänne, 2011), the emotional condition of teachers associated with GE activities has never been investigated so far. Few studies (Boler, 1999; Tallon, 2012) investigated a similar topic, namely how teachers use emotions as an educational tool in the GE context, emphasizing that altruistic emotions such as pity, compassion and guilt are frequently used to manage the class, with the possible side effect of generating forms of passive empathy unable to encourage action for social change among students.

Our study addressed this research gap by analyzing the psychological effects of being part of a common GE project on more than a thousand high school teachers from 10 European countries. In detail, the emotional states of teachers after GE activities in comparison with those experienced after standard school activities were analyzed, as well as any significant changes in their attitudes toward sustainability (in a broader sense) and migrants (as a component of intercultural sustainability relevant in contemporary school contexts, in light of the issues included in the current case study) through a pre-post experimental design. The following hypotheses are tested to identify a positive psychological impact of GE activities on teachers:

Hp. 1: After GE activities, teachers will show a decrease in negative emotions associated to teaching compared to standard lessons.

Hp. 2: After GE activities, teachers will show an increase in positive emotions associated to teaching compared to standard lessons.

Hp. 3: Positive attitudes toward sustainability will show a significant increase after GE activities, in comparison with the baseline data.

Hp. 4: Negative attitudes toward migrants will show a significant decrease after GE activities, in comparison with the baseline data.

## Method

### Participants

The current research is part of the DEAR (Development Education and Awareness Raising) European Project “Start the change!”, a GE-based project involving 12 European countries (Austria, Croatia, Czech Republic, France, Germany, Italy, Malta, Poland, Slovakia, Slovenia, Spain, United Kingdom). The aim of the project is to improve the educational offer consistently with the GE approach and contribute to the achievement of the SDGs by 2030, devoting particular attention to the relationship between climate change, migration, and global inequality. The main targets are youngsters 15–24 years old and secondary school teachers. The outcomes foresee: (1) design and implementation of educational activities on the relationship between SDGs and migrations; (2) creation/strengthening of innovative forms of youth participation in local communities; (3) building a network among schools, civic organizations, and local institutions for public awareness campaigns. The involved schools invited their teachers to attend a training phase and then to follow an applied phase to put the GE principles into practice during teaching activities focused on the issues of the project. The participation in both phases was completely voluntary, and teachers could attend the training even without implementing applied activities with students. A convenience sampling was done on the participants, only data from teachers attending both phases are considered in this study. A total of 1,311 teachers from 323 schools attended the training and 1,292 filled in the questionnaire before the attendance (98.6%), whereas 378 completed the questionnaire after concluding the activities with the students (29.3%). The current paper presents the data regarding the impact on upper secondary school teachers (ISCED 3), including teachers from 10 countries (Austria, Croatia, Czech Republic, Germany, Italy, Poland, Slovakia, Slovenia, Spain, United Kingdom), referred to a total population of 1,773,427 teachers in the European Union – 28 countries (EU-28) in 2019 (Eurostat, 2022). The average age of teachers was 43.76 years, with an average teaching experience of 14.91 years. This is consistent with data showing older populations for higher levels of education, as at ISCED 3 in EU-28 39% of teachers are aged 50 or more (Farrugia et al., 2020). The sample appears to be unbalanced by gender (82.2% women, 17.5% men, 0.3% not binary), but substantially in line with the current employment data, as according to Eurostat (2020) many of the participating countries have a percentage of female teachers in upper secondary schools between 70 and 80%.

### Procedure and Materials

To evaluate the effects of participating in the GE program, a longitudinal quasi-experimental study was designed. The GE program was coordinated by the project leader and locally implemented by the partner NGOs working with the schools and in charge of training the teachers. The program included two main phases for all the participants. The first phase was an intensive training for teachers, organized by NGOs on the issues of GE, SDGs and migration, including methodological issues for applying those principles into daily teaching; the duration of this phase was homogeneous across the countries. The second phase led them to design specific activities carried out in the following months, connecting the topics faced during the training with the specific subject taught by each teacher; the duration of this phase was subject to variations due to the school calendar of each country, yet in most cases it lasted around one school year. All the data presented in the current study are collected from teachers who completed both phases. Teachers filled in two questionnaires, the first before attending GE courses, the second at the end of their activities with students applying the GE principles. The questionnaires were administered both through paper and pencil and digital formats, according to contextual availability in the schools involved in the project. The data collection process was divided into three annual periods (2018–2020) consistent with the timing of the school years, with the first administration in the months of September-October and the second in May-June.

The measures presented through the questionnaire included:

1) Socio-demographic variables (age, gender, country of residence).

2) Emotional state when teaching, with an adapted version of the PANAS (Watson et al., 1988). The scale used for this study includes 10 words associated with positive moods (e.g., “interested; excited; strong”) and 10 words associated with negative moods (e.g., “distressed; upset; guilty”), which are rated by respondents providing an estimation of the extent they have felt that way on a 5-points Likert scale like the original version. The adaptation concerns the instructions, which invited the participants to answer thinking of the emotional state they experienced “while teaching” (“pre” condition) and “during the teaching activities, related to the training you attended, that you carried out with the students” (“post” condition). The investigation is not about the emotional state while attending the courses given by the partners involved in the project, but instead about the emotions experienced when applying those principles and teaching methods to class activities with the students. The comparison of the emotions during previous traditional teaching and this GE approach is the object of the current study. The PANAS is used to investigate emotions in teachers' population across various countries, with comparable validity and reliability values (e.g., Albuquerque et al., 2012; Buyukgoze-Kavas et al., 2014; Fernández-Berrocal et al., 2017; Li et al., 2017). Internal reliability of the scale in the current study is investigated using Cronbach's alpha (Negative emotions, 0.825 pre, 0.768 post; Positive emotions, 0.616 pre, 0.696 post). The values of negative emotions are consistent with previous studies, we accepted also values lower than.70 recorded for positive emotions relying on previous studies (Taber, 2018).

(3) Teachers' attitudes toward sustainability issues, assessed with the Attitudes Toward Sustainable Development Scale (Biasutti and Frate, 2017). It is a scale comprising 20 items which conceives sustainable development as a multidimensional construct constituted by four factors, and each of them is measured by the level of agreement with 5 items on a 5-points Likert scale. The four factors include environment (e.g., “When people interfere with the environment, they often produce disastrous consequences”), economy (e.g., Government economic policies should increase sustainable production even if it means spending more money”), education (e.g., “Teachers in college should promote future-oriented thinking in addition to historical knowledge”) and society (e.g., “Each country can do a lot to keep the peace in the world“). The scale is purposely designed to quantify the effects of educational activities consistent with sustainability principles, and the values on the four factors are expected to be sensitive to the different topics faced during the educational path. The scale is applied to investigate teachers' attitudes in different languages, showing sound validity and reliability values in the translated versions (e.g., Richter-Beuschel and Bögeholz, 2019, 2020; Andić and Curić, 2020; Echegoyen-Sanz and Martín-Ezpeleta, 2021). Internal reliability of the scale in the current study is investigated using Cronbach's alpha (Environment, 0.603 pre, 0.689 post; Economy, 0.759 pre, 0.758 post; Education, 0.756 pre, 0.792 post; Society, 0.658 pre, 0.751 post; Total, 0.844 pre, 0.862 post). The values are consistent with previous studies, we accepted also values lower than.70 according to previous studies (Biasutti and Frezza, 2009).

4) Teachers' attitudes toward migrants, with a scale adapted by Leong (2008) from the Eurobarometer Survey (Thalhammer et al., 2000). This scale includes 6 items measuring the blame toward immigrants, that is the extent to which the respondent attributes immigrants' individual and social misfortunes to themselves. The scale includes different dimensions, such as education, welfare, insecurity, criminality, treatment from the authorities, unemployment (e.g., “In schools where there are too many immigrants, the quality of education suffers”). In our perspective, such a scale offers an effective integration for the previous Attitudes Toward Sustainable Development Scale, assessing the degree of intercultural sustainability (Busch, 2016). This aspect appears to be particularly relevant considering the focus of the project and its training activities on the relationship between Sustainable Development Goals and migration, which is the core issue of the activities carried out by the partners with the teachers attending the courses. The scale is included in the Eurobarometer's studies covering 15 countries, and its reliability and validity are supported by previous studies (Thalhammer et al., 2000; Leong and Ward, 2006). Internal reliability of the scale in the current study is investigated using Cronbach's alpha (0.854 pre, 0.846 post).

All the original instruments were taken from the international literature and were written in English, hence they underwent a translation and back-translation process. The authors supervised the process with local assistants collaborating with the partner NGOs involved in the project, who were all native speakers of the target language, fluent in English and familiar with the principles of GE. For each country a first person provided the translation into the local language, which was subsequently back-translated to English by an independent translator. The comparison of the original version with the back-translations showed minor discrepancies in all languages, which were discussed by the research team involving the local assistants. Small alterations were included both in the items and the instructions, also considering the concerns regarding the clarity of the requests applied to the actual teaching context. Data were collected in an anonymous and aggregated form for statistical purposes only, in compliance with the Declaration of Helsinki and current ethical guidelines for social research provided by the Italian Psychology Association (Associazione Italiana di Psicologia). Analyses were carried out using SPSS statistical software (IBM, version 22).

## Results

To test the emotional impact of GE activities (HP 1 and HP2), a comparison was developed between the emotional state declared by teachers during standard school activities and what they experienced working with the students in the context of the GE, aimed at identifying the expected differences due to the new teaching framework. This analysis was performed through an Analysis of Variance (ANOVA).

**Figure 1:**
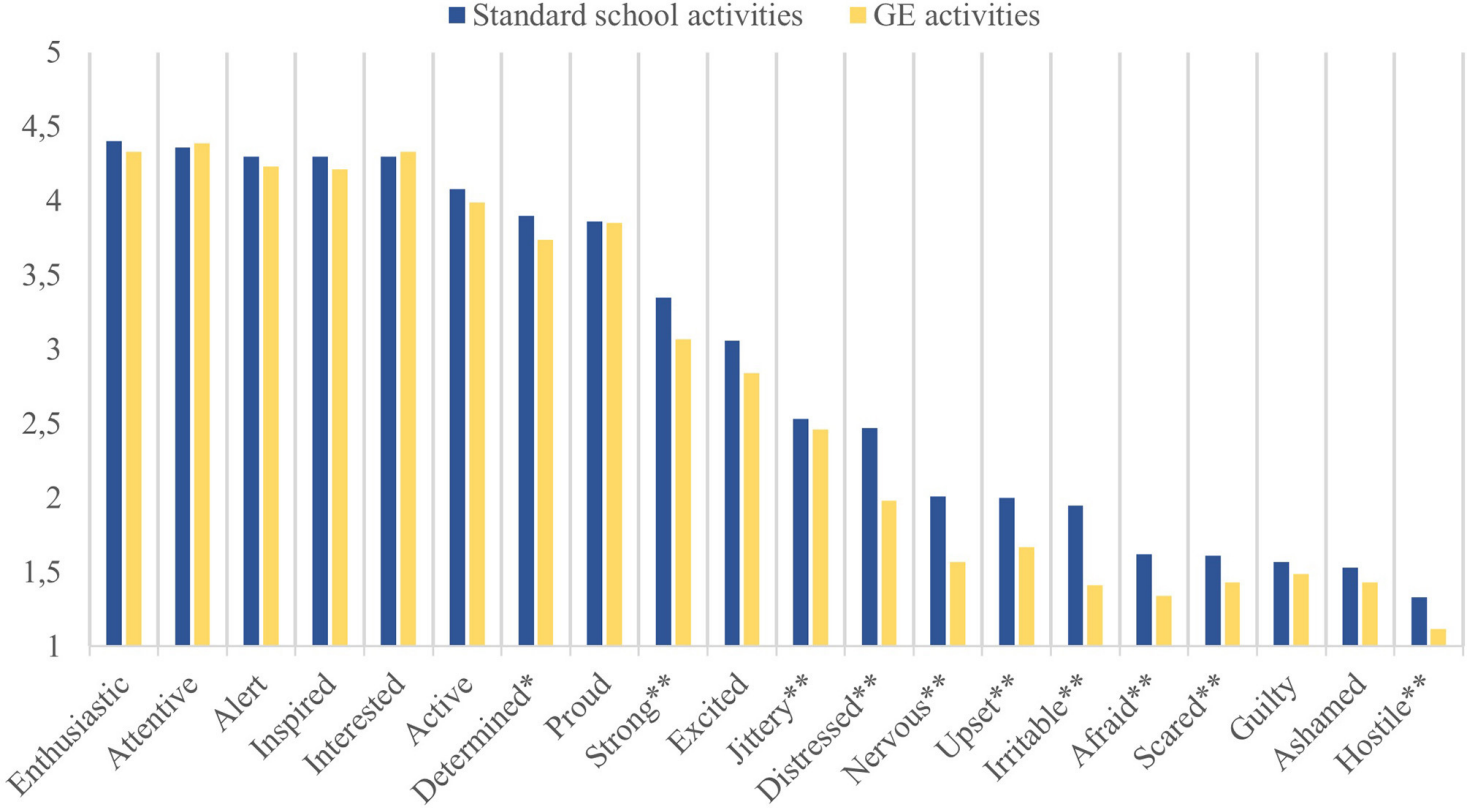
Intensity of teacher's emotions after standard school activities (blue) and Global Education (GE) activities (yellow), PANAS scale by Watson et al. (1988) (average values, *p < 0.05, **p < 0.01).

As regard to the negative emotions (HP 1), a significant reduction (p < 0.01) for many of the negative affects (hostility, irritability, distress, upset, scaredness, nervousness, feeling jittery and afraid) after GE lessons was observed when compared with standard lessons (Figure 1). In details, results showed a more intense (above 10%, Table 1) decrease in irritability (−27,69 %), nervousness (−21,89%), distress (−19,84%), being afraid (−17,28%), and upset (−16,50 %), hostility (−15,79 %) and scaredness (−11,18 %). Less intense decreases for feeling jittery (−2,77%), ashamed (−6,54%) and guilty (−5,10 %) were detected, the latter two not reaching statistical significance.

**Table 1:** Intensity of teacher's negative emotions after standard school activities and Global Education (GE) activities (average values, ^**^p < 0.01).

	**Standard school activities (m.)**	**GE Activities (m.)**	**Variation (%)**
Irritable**	1,95	1,41	−27,69
Nervous**	2,01	1,57	−21,89
Distressed**	2,47	1,98	−19,84
Afraid**	1,62	1,34	−17,28
Upset**	2	1,67	−16,50
Hostile**	1,33	1,12	−15,79
Scared**	1,61	1,43	−11,18
Ashamed	1,53	1,43	−6,54
Guilty	1,57	1,49	−5,10
Jittery**	2,53	2,46	−2,77

Addressing the positive emotions (HP 2), significant variations have been observed only for strength (*p* < 0.01, −8,36%) and determination (*p* < 0.05, −4,1 %), which were higher when referred to standard lessons. Possible age and gender effects on the emotional state of the teachers were also analyzed, to highlight any variations comparing standard school activities and GE activities. Regarding age, correlations (Table 2) showed a substantial reduction in emotional intensity (both positive and negative, R= −0.124 and −0.85 respectively, *p* < 0.01) linked to traditional school activities as teachers get older. The same relationship was found not significant when assessing the GE activities (R = −0.37 and −0.12), suggesting that older and more experienced teachers aligned themselves with their younger colleagues in mobilizing their emotional dimension according to the pattern described above. About gender, a significant difference (*p* < 0.01) between men and women in positive emotions associated with traditional school activities (higher intensity in the female gender, Table 3) was found. This same difference was not detected in GE activities, as the two genders presented the same emotional pattern. It was not possible to proceed with the statistical analysis of the data of those who identified themselves as not binary due to a limited number of cases. Concerning sustainable attitudes (HP 3), data showed an upward trend in teachers' scores after the GE program on all four dimensions. More in details (Table 4), in the pre-post comparison, the average score increased from 3.93 to 4.10 on the environmental dimension of sustainability (+4,33%), from 4.38 to 4.49 on the economic one (+2,51 %), from 3.86 to 3.90 on the social dimension (+1,04 %) and from 4.49 to 4.55 on the educational one (+1,34 %). However, this trend reached statistical significance (*p* < 0.01) for two dimensions only: environmental and economic. As already observed for emotions, the GE activities had a flattening effect on gender and age. Baseline correlation data showed indeed a significant increase in sustainable attitudes with older age on two sub-factors, namely environmental and economic sustainability (Table 5). Conversely, analyzing the data collected at the end of the GE activities no further differences related to the teachers' age were detected. Furthermore, Table 6 shows that not only the average dispositions toward sustainability increased in both genders in the pre-post comparison, but also that the difference previously detected (p < 0.01) on the dimension of educational sustainability disappeared, whereas a variation on the economy factor emerged.

**Table 2:** Correlations between teachers age and the intensity of their positive/negative emotions after standard school activities and Global Education (GE) activities (R, **p < 0.01).

	**Standard school activities**		**GE activities**	
	**Positive emotions**	**Negative emotions**	**Positive Emotions**	**Negative emotions**
**Teachers' age**	−0.124**	−0.85**	−0.37	−0.12

**Table 3:** Correlations between teachers' genders and the intensity of their positive/negative emotions after standard school activities and Global Education (GE) activities (average values, ^**^p < 0.01).

	**Positive Emotions**		**Negative Emotions**	
	Standard school activities**	**GE activities**	**Standard school activities**	**GE activities**
Men	3,28	3,15	1,68	1,41
Women	3,52	3,28	1,73	1,41

**Table 4:** Intensity of teacher's attitudes toward sustainability before and after their Global Education (GE) activities (average values, ^**^p < 0.01).

	**Before GE activities**	**After GE activities**	**Variation (%)**
Environmental**	3,93	4,1	4,33
Economic**	4,38	4,49	2,51
Educational	4,49	4,55	1,34
Social	3,86	3,9	1,04

**Table 5:** Correlations between teachers' age and their scores on the attitudes toward sustainable development scale before and after their Global Education (GE) activities (R, **p < 0.01).

	**Environment**		**Economy**		**Society**		**Education**	
	**Before GE activities**	**After GE activities**	Before GE actiities**	After GE actiities**	Before GE actiities**	**After GE activities**	**Before GE activities**	**After GE activities**
Teachers' age	−0.12	0.15	−0.95	0.90	0.71	−0.34	−0.50	−0.30

**Table 6:** Teachers' gender and their scores on the attitudes toward sustainable development scale before and after their Global Education (GE) activities (average values, **p < 0.01).

	**Environment**		**Economy**		**Society**		**Education**	
	**Before GE activities**	**After GE activities**	**Before GE activities**	After GE activities**	**Before GE activities**	**After GE activities**	Before GE activities**	**After GE activities**
Men	3,9	4,13	4,38	4,62	3,84	3,96	4,41	4,54
Women	3,94	4,05	4,38	4,45	3,87	3,9	4,52	4,54

**Figure 2:**
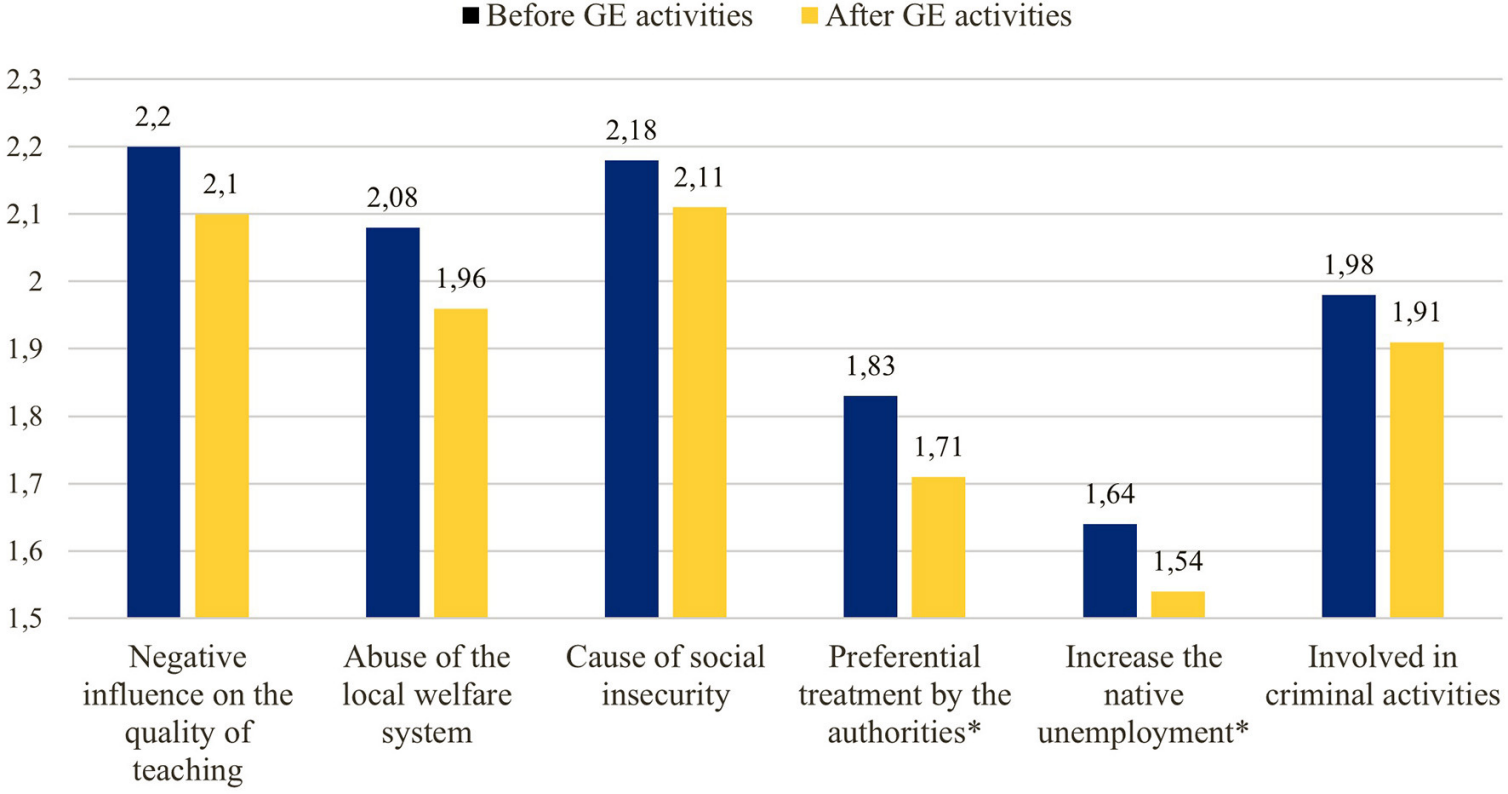
Intensity of teachers' negative attitudes toward migrants before (blue) and after (yellow) Global Education (GE) activities, scale by Leong (2008) (average values, *p < 0.05).

Finally, negative attitudes toward migrants were also compared, testing HP 4 (Figure 2 and Table 7). The expected reduction in negative attitudes was found. The general average scoring of the adopted scale showed a significant decrease (*p* < 0.5, from 1.98 to 1.88). Considering the single items, significant changes (*p* < 0.5) on two beliefs were detected (“Migrants have a preferential treatment by the authorities,” −6,56 %, and “Migrants contribute to increase unemployment among natives,” –6,09 %).

**Table 7:** Intensity of teachers' negative attitudes toward migrants before and after their Global Education (GE) activities (average values, ^**^p < 0.01).

	**Before GE activities**	**After GE activities**	**Variation (%)**
Negative influence on quality of teaching	2,22	2,10	−5,41
Abuse of the local welfare system	2,08	1,96	−5,77
Cause of social insecurity	2,18	2,11	−3,21
Preferential treatment by the authorities**	1,83	1,71	−6,56
Increase the native unemployment**	1,64	1,54	−6,09
Involved in criminal activities	1,98	1,91	−3,54

Moreover, a significant correlation (R = 0.71; *p* < 0.05) between age and negative attitudes toward migrants was found analyzing the baseline data, as prejudices toward them increased as teachers get older. Conversely, no differences due to age were detected after the GE activities, highlighting that older teachers were subject to the same decreasing trend noticed above. No significant variations by gender were observed.

## Discussion

Results showed that the applied GE activities had significant positive effects on all the addressed variables: emotions associated with teaching, attitudes about sustainability, understood as a multidimensional concept, and the mitigation of negative attitudes toward migrants.

As regard to HP 1, it was found to be fully verified as significant decreases compared to standard school activities were observed for a wide range of negative emotions. It implies that GE activities have had a general impact in reducing the usual teaching stress, and an even stronger influence on more momentary and context-dependent emotions (e.g., irritability, nervousness). These findings present wider implications, as psychological stress and burnout in teachers are a social problem that impacts on their personal health, on the quality of their job performances and on students' education (i.e., Klusmann et al., 2008; Saloviita and Pakarinen, 2021). Also, as pointed out by Herman et al. (2020), most teachers present a latent profile characterized by a high stress coupled with high (66%) or low (28%) coping skills, whereas only 6% of them experience low stress levels at work. It can therefore be argued that spreading GE-based activities at school would have a significant impact on the balance between stress levels and coping skills, decreasing the extent of perceived stress, and positively affecting the actual incidence of burnout and mental health problems. Moreover, according to Koenen et al. (2019), the presence of less intense negative emotions can be related to a greater sensitivity toward students, as well as fostering higher levels of engagement during classroom activities (Burić and Macuka, 2018). More in general, a decrease in teachers' negative emotions constitutes a positive factor for their educational performances, as negative emotions have been often found to be related with diminished levels of cognitive functioning and motivation while teaching (Sutton and Wheatley, 2003). Concerning age, a substantial leveling effect for GE activities was detected. This allows us to infer that the emotional differences related to individual experience (i.e., years of teaching), characterizing standard classroom activities, are not present for GE activities. These latter activities could therefore be a factor in promoting a more egalitarian psychological experience, at least as regards negative emotions, favoring fruitful forms of intergenerational dialogue between teachers.

Although GE activities have had a significant impact reducing negative emotions linked to teaching, the same cannot be said for positive ones, which remained stable in most cases. The HP 2 is therefore not confirmed. This result is not entirely surprising if it is considered that teachers declared experiencing a high average level of positive emotions associated with standard school activities too, and that many of the measured affects evoked a high mobilization of psychophysical abilities for performance purposes, such as alertness, determination, and strongness.

Considering the results on emotional states, it can be concluded that GE activities show a significant decrease in the intensity of negative affects without any impact on most of the positive affects, which maintain the high intensity experienced by teachers linked to standard school activities. It must be further investigated if this is a prerequisite for promoting optimal experiences among teachers, as the resulting emotional pattern emerged after GE activities is consistent with a better balance between environmental challenges and perceived skills which is one of the features of flow experience.

Addressing HP 3, results proved its consistency, as an upward trend in attitudes toward sustainability was found after GE activities in comparison with the baseline data. Remarkably, this trend is established despite the high baseline values of teachers' attitudes, attesting that GE activities can promote further development of sustainable consciousness even where it is already well-developed. Those findings also highlighted that school activities based on the GE framework can significantly impact on sustainable attitudes as previously demonstrated for similar educational approaches with teachers, namely Education for Sustainability (Merritt et al., 2019) or Education for Sustainable Development (Andersson et al., 2013; Nousheen et al., 2020; Braßler and Sprenger, 2021). As higher sustainable attitudes have been generally found to reinforce the subjective pro-environmental behaviors (Gifford and Sussman, 2012) and, in the case of teachers, affect in turn their students' values (Murphy et al., 2020), it can be argued that participating in GE activities could have positive effects on the sustainable behavior of the teachers themselves and on their students' sustainable awareness. Data also suggested that the pre-post difference was significant only on two subscales: environmental and economic sustainability. This may have been due to the thematic focus of the project on inequalities and their environmental effects, but the multiplicity of the applied activities does not allow a linear verification of this hypothesis, which should be tested in future research. Regarding age, results suggested a stronger focus of older teachers on economic sustainability after GE activities, as well as a leveling effect on the social one. As for gender, GE activities exerted a major focus on economic sustainability was detected in males after GE activities, whereas this latter showed an equalizing effect on the importance that men and women teachers attributed to educational sustainability.

Concerning HP4, it was fully verified, as a significant decrease of negative attitudes toward immigrants within teachers was observed after GE activities, in comparison with the baseline. As also noted above, this result occurs even though the average level of prejudice in teachers was already low at the beginning. Participation in GE-based activities was therefore found to be effective in reducing negative attitudes toward migrants in a target, such as that of teachers, who already had a strong awareness on this topic. More generally, as to fight against the explicit and implicit forms of racism and discrimination at school is a crucial issue in today's multicultural societies (Farkas and Gergely, 2020) our results could support the extensive use of GE-based training programs for teachers, as their efficacy on promoting a decreasing in prejudice was tested.

## Conclusions

The findings of the current article offer a strong support for the application of GE principles, yet they are subject to some limitations. In the first place there is no control group as the project did not provide the chance of collecting data from a homogenous population not involved in GE activities for the sake of comparison. This offers the possibility of reasoning about correlations between the selected variables, but without knowing if the observed effects are due specifically to the GE principles or other experimental conditions. The results might be attributable also to the well know Hawthorne effect in this case study, as the effort put by the employer school in offering training activities, the involvement of external actors (e.g., NGOs) and the direct connection with applied activities are relevant factors that may influence the experience of the teachers. This limitation is shared with many studies on the impact of similar approaches (Nousheen et al., 2020; Braßler and Sprenger, 2021), as the many intervening variables (e.g., organizational, didactic, and social) generally hinder the use of control groups in the school environment. Strengthening the collaboration between school institutions in charge of the didactic and organizational activities and actors carrying out assessment and analysis would offer a better comprehension of alternative educational approaches. Moreover, desirability biases and compliance with the observers may have influenced teachers answers, especially considering the sensitive issues at stake; despite this, we argue that a partial reduction of such effect was achieved by using self-administered surveys instead of interviews and guaranteeing the anonymity of respondents by not providing schools (or local project representatives) with direct feedback on their teachers' responses. One of the most relevant improvement of the current study implies a further investigation including teachers maintaining the traditional activities with students, as it would offer the opportunity for a direct comparative assessment which can lead to a better appraisal of GE approach effects. In the second place, teachers' emotional states were measured at the end of the teaching period, through deliberate answers to questions investigating the memory of the overall experience which lasted several months. The necessary mental elaboration for such a request can increase the distance between the momentary emotional state and its recollection. Applying a research procedure like the Experience Sampling Method (ESM), which collects systematic answers at random times during the daily lives of participants (Larson and Csikszentmihalyi, 2014), is a fruitful integration to the approach developed in the current study. Moreover, the opportunities offered by wearable digital devices can extend the analysis to psychophysiological measures, providing real-time, not voluntarily controlled, and non-invasive assessment of teachers' personal experience. Combining such information is considered a more complete representation of subconscious and conscious emotions (Chamberlain and Broderick, 2007). In the third place, the large sample size included a heterogeneous population which varied according to a range of variables; despite all the activities were designed by the partners under the coordination of the project leader and inspired by common GE principles, local differences affected the actual implementation of the activities (e.g., locally relevant topics, activities' locations, peculiar ways in which the GE methodology has been integrated into the subject of teaching). Observations conducted in a more homogenous sample can facilitate controlling for such variables. Finally, national differences have not been addressed in this article, but it is reasonable that they may represent an element of variability among teachers when emotional dimensions and sustainable attitudes are concerned. Therefore, future research would investigate the cultural differences between teachers on a European or international scale and how these could impact on the effectiveness of GE programs, possibly reducing other intervening variables.

Notwithstanding such limitations, these results suggest some reflections on GE approach and its assessment. In general, the data provide sound support to the transformative potential of GE for teachers, as the affective state is positively influenced by the training and the actual implementation of such principles in daily teaching practice. These also positively modify different dimensions of sustainability, namely environmental, economic and intercultural. Although a significant change is not observed for all the assessed sustainability dimensions, results are consistent with the declared GE principles and the wider SDGs framework which encourage a holistic approach. Moreover, although teachers of different age and gender experience diverse emotional reactions after traditional teaching activities, GE activities are associated with more homogenous emotional patterns across the different socio-demographic populations. This implies that GE approach can be conceived as a fruitful protective factor against work-related stress for teachers, not only because it diminishes negative emotions, but also thanks to its capability of inducing a more uniform affective state in teachers' population. Having a more positive emotional experience while teaching and perceiving it as a common state shared with colleagues can foster teachers' wellbeing.

The relevance of these results is twofold, as they offer new insights both on the theoretical and practical level. Theoretically they fill the gap about the psychological impact of GE on teachers, thus completing the framework offered by previous research about teachers' needs emerging from traditional approach to teaching (Mundy and Manion, 2008; Reysen and Katzarska-Miller, 2018) and motivations supporting the adoption of GE principles (Biberman-Shalev, 2021). Practically, it suggests that fruitful constructs can be identified from the literature, whose selection should be partly based on the specific contents of the teaching courses and the objectives of the assessment for all the actors involved. In general, notwithstanding the possible differences resulting from the theoretical and methodological choices made, we consider the psychological assessment approach as a fruitful integration in this kind of teaching initiatives to provide insights about their impact. Such type of assessment allows scholars and professionals to go beyond the mere description of a phenomenon, for example recording the satisfaction for a specific course; it opens the way for making hypotheses on the mental mechanisms underlying the efficacy of a teaching approach, strongly connecting the results with the literature on the selected constructs.

Yet, when it comes to defining some guidelines for implementing GE initiatives, the issue of measurement must not be conceived only as an assessment tool, emphasizing the usage of data for an estimation of the performance of a training activity. Certainly, it can be a useful support for comparing different approaches, methodologies, school subjects, and for providing sound quantitative support to other qualitative inquiries. However, in addition to this knowledge about performance it can be a powerful tool for self-reflection of teachers, providing detailed feedback on the results attained as a personal and professional growth in line with the opportunities offered by ESM method. This can inform the individuals and the institutional actors involved in the process and in charge of making decisions about the design and implementation of new teaching initiatives. The ultimate goal is to make them more aware of the importance of integrating assessment tools as a fundamental element of the design phase, broadening the field of observation including not only the technical competences acquired but also the personal transformation taking place and its perceived quality. Indeed, such psychological change can account for a long-lasting transformation of teachers and their behavior. Such approach would offer policy makers and school professionals (e.g., teachers and principals) theoretical and practical tools to be better equipped with evidence-based findings to face the challenges of education in a global age (Ramkissoon, 2022). The conception of education both as a “subject and object” of change (Marginson, 1999, p. 20) assigned a greater responsibility to teachers in developing a sense of citizenship by means of curricula and pedagogical choices (Vongalis-Macrow, 2004). These local education policies interact with the national level, which in turn deals with a global agenda for education (Edwards, 2017): the resulting field includes both international actors (e.g., multilateral institutions, foreign aid agencies, international NGOs, transnational corporations, consultancy companies, and philanthropic foundations) and national actors (e.g., policymakers, governmental agencies, national NGOs) (Ball, 2012; Lingard et al., 2015). This complex scenario involves different approaches to education policies and calls teachers to find a balance between global and local interests. Conceiving psychological assessment as both a performance and self-reflection tool can supply teachers with a reference to deal with such complexity, supporting them in comparing heterogenous educational contexts and in developing effective coping mechanisms in the long term (Ramkissoon, 2021).

The results must be conceived as a platform for developing future research on the GE approach to teaching. In addition to the further research suggested to overcome the limitations of the current study, other directions of investigation can be considered. A direct comparison between GE and other approaches suggested by the literature would be beneficial, to identify the most fruitful theoretical frameworks to strengthen the impact of education in reaching SDGs. Moreover, a closer classification of enacted educational practices (e.g., educational tools, length of the program activities, actors involved in addition to school teachers, subjects taught) could shed light on how effective the integration of GE principles is in daily school activities. The psychological variables should also be complemented by behavioral variables, regarding for example school activities (e.g., the availability to take responsibility for other school duties of tutorial nature, or to engage into multidisciplinary projects) and other non-strictly educational tasks (e.g., involvement in civic duties). This would help in understanding the impact of the psychological variables on the actual behavior, focusing on the specific population of teachers. These types of observation should also be extended to students, offering empirical support to the final impact of GE on young generations and the actual transfer of the positive effects observed among teachers to the wider population of students.

## Data Availability Statement

The datasets presented in this article are not readily available because of the agreements among the partners involved in the project. Requests to access the datasets should be directed to marco.boffi@unimi.it.

## Ethics Statement

Ethical review and approval was not required for the study on human participants in accordance with the local legislation and institutional requirements. The patients/participants provided their written informed consent to participate in this study.

## Author Contributions

MB: conceptualization, methodology, investigation, data curation, and writing–review and editing. NR: conceptualization, methodology, formal analysis, investigation, data curation, and writing–original draft. PI: supervision. All authors contributed to the article and approved the submitted version.

## Conflict of Interest

The authors declare that the research was conducted in the absence of any commercial or financial relationships that could be construed as a potential conflict of interest.

## Publisher's Note

All claims expressed in this article are solely those of the authors and do not necessarily represent those of their affiliated organizations, or those of the publisher, the editors and the reviewers. Any product that may be evaluated in this article, or claim that may be made by its manufacturer, is not guaranteed or endorsed by the publisher.
